# Dynamic and Static Assembly of Sulfated Cellulose Nanocrystals with Alkali Metal Counter Cations

**DOI:** 10.3390/nano12183131

**Published:** 2022-09-09

**Authors:** Patrick Petschacher, Reza Ghanbari, Carina Sampl, Helmar Wiltsche, Roland Kádár, Stefan Spirk, Tiina Nypelö

**Affiliations:** 1 Institute of Bioproducts and Paper Technology, Graz University of Technology, Inffeldgasse 23, 8010 Graz, Austria; 2 Department of Industrial Materials Science, Chalmers University of Technology, 41296 Gothenburg, Sweden; 3 Institute of Analytical Chemistry and Food Chemistry, Graz University of Technology, 8010 Graz, Austria; 4 Department of Chemistry and Chemical Engineering, Chalmers University of Technology, 41296 Gothenburg, Sweden; 5 Wallenberg Wood Science Center, Chalmers University of Technology, 41296 Gothenburg, Sweden

**Keywords:** cellulose nanocrystals, ion exchange, ion pairs, rheology, birefringence, rheo-PLI

## Abstract

Sulfate groups on cellulose particles such as cellulose nanocrystals (CNCs) provide colloidal stability credit to electrostatic repulsion between the like-charged particles. The introduction of sodium counter cations on the sulfate groups enables drying of the CNC suspensions without irreversible aggregation. Less is known about the effect of other counter cations than sodium on extending the properties of the CNC particles. Here, we introduce the alkali metal counter cations, Li^+^, Na^+^, K^+^, Rb^+^, and Cs^+^, on sulfated CNCs without an ion exchange resin, which, so far, has been a common practice. We demonstrate that the facile ion exchange is an efficient method to exchange to any alkali metal cation of sulfate half esters, with exchange rates between 76 and 89%. The ability to form liquid crystalline order in rest was observed by the presence of birefringence patterns and followed the Hofmeister series prediction of a decreasing ability to form anisotropy with an increasing element number. However, we observed the K-CNC rheology and birefringence as a stand-out case within the series of alkali metal modifications, with dynamic moduli and loss tangent indicating a network disruptive effect compared to the other counter cations, whereas observation of the development of birefringence patterns in flow showed the absence of self- or dynamically-assembled liquid crystalline order.

## 1. Introduction

Cellulose nanocrystals (CNCs) are among the few biological nanomaterials which are industrially manufactured [1]. A common manufacturing process involves sulfuric acid that esterifies the CNCs being decorated with sulfate half ester groups [2]. The counterions of the sulfate groups are decisive for the material properties of CNC water suspensions. Usually, the suspensions experience critical concentrations below which they exhibit isotropic ordering, and above which they arrange in liquid crystalline assemblies [3]. In water suspensions, protonated sulfated CNCs have been reported to form liquid crystalline orders at lower concentrations than suspensions where the counter ion is a sodium, potassium, or a cesium cation. The critical concentration for ordered phase formation increased in the order H^+^ < Na^+^ < K^+^ < Cs^+^ [4]. The protonated CNCs form nematic ordering in the suspension, whereas the Li-CNC suspension is isotropic [5]. This suggests that controlling the ordering of CNCs in water suspensions may be realized by the choice of counterion. Assembling CNCs in nematic and unidirectional hierarchies has been in the focus for facilitating optical effects via birefringence (glitter [6]) to realize, e.g., structural colors [7,8,9], photonic effects [10,11], and optical sensing [12].

The proton or cation exchange has been addressed in the context of sulfate group content determination [13,14], where for the determination by conductometric titration, a conversion of the Na-grade into the acidic form is required. The extent of the exchange achievable by dialysis has been reported to be incomplete [14,15], and, hence, conversion to the acidic form using ion exchange resins is common [14]. However, the use of ion exchange resins is challenging at higher concentrations of hydrophilic nanoparticles because they retain water, and the concomitant viscosity increase complicates the transport in the resin matrix.

Counterion exchange to the H and Na form is trivial, whereas fewer contributions address other monovalent cations. The conversion to Na, K, and Cs forms was performed by neutralizing the acid form suspension with the corresponding base until its pH was neutral [4]. However, this procedure first passes the Na-CNC suspension through an ion-exchange resin column to provide the protonated form for further conversion [16]. The ion-exchange process converts Na-CNC suspensions into H-CNC, and lowers the pH from 7 to about 3. K-CNC was obtained by adding 1 mM potassium hydroxide to the H-CNC suspension until a pH value of 6.6 was reached [16].

Here, we demonstrate a methodology for the ion exchange of Na-CNC to the alkali metal monovalent cations without using ion exchange resins. We hypothesize that the sulfate groups can be ion-exchanged in a similar manner as carboxylic acid groups [17] by adjustment of the pH above the pK_a_ value and exposure to the excess of the exchangeable cation/proton. The pK_a_ value of sulfate ester groups is around 2 [13], indicating that for exchanging the proton to a cation, the pH above the pK_a_ should be used to allow replacement of the protons. Although the exchange should be already possible at the pK_a_, where theoretically 50% of the groups are dissociated, the pH at the surface is different from the bulk. As there are more protons at the surface than in the bulk, a higher pH value than the pK_a_ may be required. We further hypothesize that, as implied by the Hofmeister series categorizing the alkali metal cations into water structure making (kosmotropic) and water structure breaking (chaotropic), the choice of the counter cation can be used to steer the flow properties of aqueous CNC suspensions. We direct the findings of our investigation for the communities dealing with the fundamentals of the cellulose functionalities, as well as applied research that exploits the surface properties, and colloidal and nanoparticle features of cellulose nanocrystals.

## 2. Materials and Methods

Cellulose nanocrystals were purchased as powder from Celluforce (NCC^®^, Quebec, Canada). Lithium chloride (LiCl) was purchased from Carl Roth (Karlsruhe, Germany) and used as received. Potassium chloride (KCl) and sodium hydrogen carbonate (NaHCO_3_) were purchased from Merck (Germany) and used as received. Rubidium chloride (RbCl), cesium chloride (CsCl), and hydrogen peroxide (H_2_O_2_, 30% in water) were purchased from Sigma Aldrich and used as received. Sulfuric acid (H_2_SO_4_, 95%) was purchased from Fisher Scientific and used as received. High-purity water from a Barnstead Nanopure water purification system (ThermoFisher Scientific, Waltham, MA, USA) with resistivity of 18.2 MΩ/cm, or water (VWR, Vienna, Austria) containing the following amounts of the ions that are relevant to this study were used: Cl < 0.05 mg/L, K < 0.005 mg/L, Li < 0.002 mg/L, Na < 0.01 mg/L, SO_4_ < 0.1 mg/L. Sodium hydroxide (NaOH) and hydrochloric acid (HCl, 37%) were purchased from VWR and used as received. Silicon wafers were purchased from Siegert Wafers (Germany) with a wafer thickness 675 ± 25 μm.

### 2.1. Preparation of CNC Suspensions and Counter-Cation Exchange Procedure

CNC powder was dispersed in water to achieve a concentration of 2 wt.%. The suspension was stirred overnight and was sonicated (Sonorex TK 52 H, 120 W) for one hour, and additionally shaken by hand every fifteen minutes. Afterwards, the suspension was cooled to room temperature and was adjusted to a pH value 2 using HCl to achieve protonation. The protonated suspension was stirred continuously for one hour. The suspension was dialyzed in water for seven days to remove unbound ions. For cation exchange, to prepare Li-CNC, Na-CNC, K-CNC, Rb-CNC, and Cs-CNC, 30 g of H-CNC was treated with LiCl, NaHCO_3_, KCl, RbCl, and CsCl, respectively. The amount of salt added was based on the sulfur content of CNC per gram, which was determined by elemental analysis, as well as the molecular weight of the salt. To guarantee a successful exchange of ions, the amount of salt exceeded the sulfur content by ten times. After the addition of salt, the suspension was neutralized by adding 0.1 M NaOH, and stirred for one hour before dialyzing it for seven days in water. The final suspensions were stored in a fridge. The concentration of the suspensions was determined gravimetrically. After the dialysis, the average concentrations of the cation-exchange suspensions were 0.97 ± 0.05 wt.%.

### 2.2. Atomic Force Microscopy

Atomic force microscopy (AFM) images were collected in tapping mode at ambient conditions with a Tosca™ 400 atomic force microscope (Anton Paar, Graz, Austria) utilizing silicon cantilevers (AP-ARROW-NCR) from Nanoworld AG (Neuchâtel, Switzerland) with a force constant of 42 N m^−1^ and a resonance frequency of 285 kHz. Image processing was accomplished with Tosca™ analysis software and Gwyddion 2.56 software.

### 2.3. Elemental Analysis

The concentrations of Li, Na, K, and S were determined by axially-viewed, inductively-coupled optical emission spectrometry (ICP-OES; Ciros Vision EOP, Spectro, Kleve, Germany). The specimens were prepared via microwave-assisted digestion: 100 mg of dry CNC powder was digested with 4 mL HNO_3_ (concentrated; purified by subboiling) and 2 mL HCl (Suprapur, Merck, Darmstadt, Germany) at 200 °C and 20 bar using a Multiwave GO (Anton Paar, Austria) microwave-assisted sample digestion system. The temperature program consisted of a 20 min ramp starting from room temperature to 200 °C. This temperature was then maintained for 10 min. After cooling to room temperature, the clear digests were made to a final volume of 50 mL. The analyte quantification was performed using emission lines, where Li (I), 670.780 nm; Na (I), 589.592 nm; K (I), 766.491 nm; and S (I), 180.731 nm. Scandium was used as internal standard at a concentration of 1 mg/L, employing the Sc (II) 361.384 nm emission line. The calibration range for the alkaline elements was 0.05 to 1 mg/L and 1 to 40 mg/L for S. Lacking detector coverage on the ICP-OES instrument, Cs and Rb were quantified by inductively-coupled plasma mass spectrometry (ICP-MS, Elan DRCe, PerkinElmer, Waltham, MA, USA). Indium was used as internal standard at a concentration of 10 µg/l. The calibration range was 0.1 to 10 µg/L.

The Na^+^/S ratio was calculated based on the elemental analysis. For this purpose, the moles (Na and S) per kg were calculated from elemental analysis, and the ratio was taken. In the same manner, the sodium content of the ion-exchanged CNC suspensions was calculated. The moles of Na^+^ per kg sample were divided by the sum of the moles of all cations (Li^+^-Cs^+^) present in one kg.

### 2.4. X-Ray Photoelectron Spectrometer (XPS) PHI VersaProbe III

XPS analysis was performed using a PHI 5000 VersaProbe III Scanning XPS Microprobe (Physical Electronics, Feldkirchen, Germany) at an angle of 45°. The CNC suspensions were drop casted on the silicon wafers, dried, and subjected to the XPS analysis.

### 2.5. Dynamic Light Scattering (DLS)

DLS measurements were performed using a Litesizer^TM^ 500 (Anton Paar, Graz, Austria) with a wavelength of 658 nm at a temperature of 20 °C. The CNC dispersions of 0.050 wt.% and 0.025 wt.% were measured in disposable cuvettes. The hydrodynamic radius is derived from the Stokes–Einstein equation for spherical particles, leading to the result of CNC to be an estimation of the apparent size of the hydrated nanocrystals or their clusters. Data acquisition was performed with Kalliope™. The analysis was performed in three repetitions.

### 2.6. Rheology

An Anton Paar MCR 702 Twin Driver rheometer (Graz, Austria) with a single motor-transducer configuration, based on a P-PTD200/GL (GL-glass) measuring cell and a parallel plate (PP)/GL geometry with a (2R =) 43 mm diameter at a measuring gap of 1 mm, was employed for measuring the steady shear viscosity of the CNC suspensions. Oscillatory shear strain sweep tests were conducted with a similar configuration using a (2R =) 50 mm diameter PP geometry at the measuring gap of 1 mm. The strain sweep tests were conducted within a strain amplitude range of 10^−3^ to 10^3^% at a constant angular frequency of *ω* = 6.28 rad⁄s.

### 2.7. Rheology-Polarized Light Imaging

The setup used for the steady shear viscosity measurements described above was augmented with a custom polarized light imaging (PLI) system for simultaneously recording the viscosity functions and polarized optical visualizations of the flow, i.e., rheo-PLI. Two linear polarizers with a 45° relative orientation were situated above and below the PP geometry, respectively, for observing the birefringence patterns. A Canon DSLR setup with a Canon L-series 100 mm macro lens (Tokyo, Japan) coupled with a Canon 25 mm extension tube was used to perform HD video recordings (1280 × 720 px) at a frame rate of 60 fps from below. The visualization setup is described in greater detail elsewhere [18,19,20].

## 3. Results

### 3.1. Introducing Alkali Metal Cations to the Sulfated CNCs

**Figure 1:**
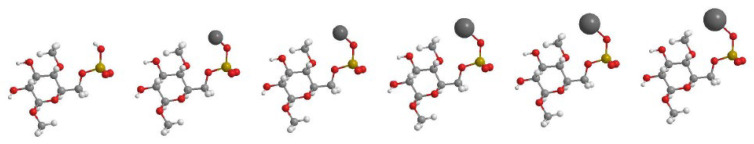
Illustration of the structure of cellulose anhydroglucose unit (AGU) with sulfate half ester group with H^+^, Li^+^, Na^+^, K^+^, Rb^+^, and Cs^+^ counter ion. The illustration was prepared using Chemdraw and is not a result of modeling.

The size of the ions in the first group increases with the increasing atomic number in the order Li^+^ < Na^+^ < K^+^ < Rb^+^ < Cs^+^, whereas the degree in the cation’s hydration decreases. The increase in cation size leads to an increase in the size of the sulfate half ester group with the corresponding counter cations (Figure 1). Furthermore, the decrease in degree of hydration leads to the low atomic number cations being structure-making and the higher number structure-breaking cations, referring to their hydrating or dehydrating capability (so-called Hofmeister series). The alkali metal counter cations are more electropositive than the sulfur, leading to an electron-rich sulfur atom. As electronegativity of the alkali metals decreases with an increasing atomic number, the electropositivity on sulfur increases, hence enhancing the stability of the sulfate group. 

The stock CNC suspension contained approximately 0.74 wt.% of sulfur based on elemental analysis (Table 1). This means that approximately every 26th AGU is substituted with the sulfate half ester group, which is an estimate based on comparing the amount of sulfur to the total cellulose amount. Since sulfate groups are assumed to reside only on the CNC surface as a result of CNCs being impermeable to the acid-catalyzed hydrolysis reaction, the approximation reflects the entire bulk mass ratio rather than the situation at the surface of the CNCs. The factual surface coverage of CNCs with sulfate half ester groups is higher. In previous work, approximately 15% of the surface functional groups were determined to be sulfate half ester groups, based on similar elemental composition [21].

The cation exchange to the sulfate half ester groups takes place on the CNC surface where these functional groups reside. The treatment of the CNCs with HCl that was targeted to protonate the sulfate groups to acidic form did not lead to a reduction of the sulfur content (Table 1), hence indicating that the sulfate half ester groups were retained during the protonation. Further treatment of the H-CNC with the alkali metal cation salts led to CNCs that retained the sulfur content, on average, at 0.71 wt.%, which corresponds to every 28th AGU to be substituted (bulk-mass ratio).

**Figure 2:**
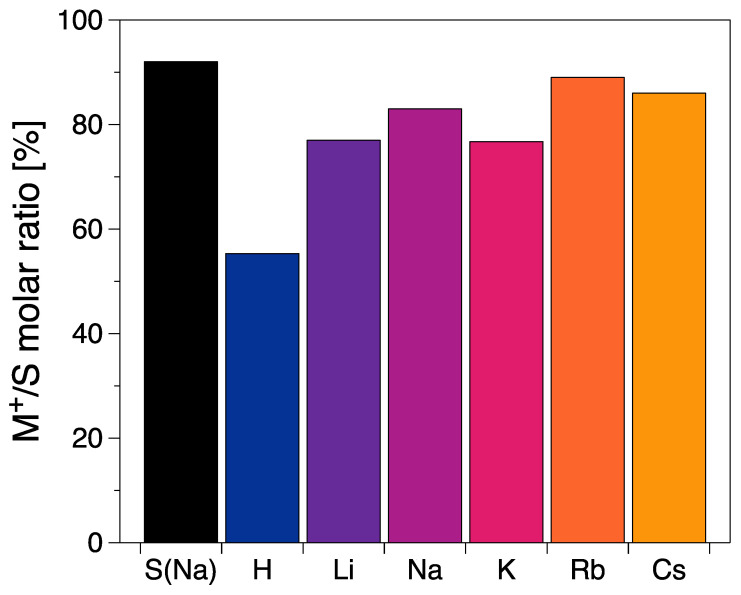
Molar ratio between the corresponding metal cation (M^+^/S) and sulfur introduced on the CNCs.

The CNC stock suspension was prepared using a commercial product that was delivered as a powder. The stock suspension contained approximately 4.9 g/kg sodium (Table 1). Using the molar mass of sodium (22.990 u) and sulfur (32.065 u), a Na^+^/S molar ratio of 0.92 is obtained. The conversion of the stock CNCs into H-CNC was accomplished using HCl for the protonation. Although it produced a mainly protonated form, a considerable amount of sulfate groups (ca. 40%) was still not exchanged. This indicates that the conversion of the commercial Na-form into the acid from (H-form) is not straightforward, and the use of ion-exchange resins is required when full conversion is a necessity, for example, to enable accurate conductometric titration [14]. However, the conversion of the H-CNC back to the Na-CNCs by providing excess sodium resulted in CNCs with nearly complete conversion (molar Na^+^/S ratio 0.83). Li-CNCs had the lowest M^+^/S ratio (0.77), whereas Rb-CNC and Cs-CNC had the highest ratios (0.89 and 0.86, respectively (Figure 2)). This analysis indicates that the ion exchange is not only possible from the protonated, but also from the sodium form. In all these grades, sodium ions are also present (Li-CNC: 12.6, K-CNC: 7.0, Rb-CNC: 7.9, and Cs-CNC: 6.6 mol.%), whose concentration was calculated from the elemental composition presented in Table 1. 

The atomic composition, determined using XPS (Supporting Information Table S1), confirmed the elemental composition by the ICP-OES (Table S1), and additionally indicated that chlorides were not present in the modified CNCs. However, the low amounts of the cations and the sulfur (<0.4 at.%), and the differentiating susceptibility of the elements, impede a thorough quantitative analysis of the spectra (hence, Li-CNC is not presented). For instance, the concentration of residual sodium in the exchanged suspensions cannot be determined due to its low concentration. However, XPS enables to assess the chemical environment of the surface species. As XPS is sensitive in chemical bonding, the metal ions experience a similar coordination environment in the metal ion modified CNCs (M-CNCs) as in the pure sulfates. The binding energies of the metal peaks in the XPS spectra of the corresponding metal sulfates and the ion-exchanged sulfated CNCs are in a good agreement (Table S2) [22].

**Figure 3:**
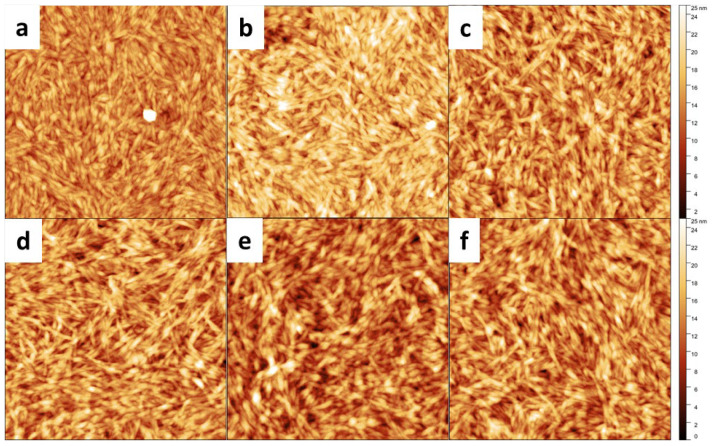
Atomic force microscopy topography imaging (2 × 2 µm^2^) of casted films using suspensions of (**a**) H-CNC, (**b**) Li-CNC, (**c**) Na-CNC, (**d**) K-CNC, (**e**) Rb-CNC, and (**f**) Cs-CNC.

The appearance of the CNCs did not change by the cation exchange, as evidenced by AFM imaging of the CNCs on a support (Figure 3). We also analyzed the hydrodynamic diameter in the 0.050 and 0.025 wt.% suspensions; however, no trend of occupied space taken by the CNCs could be detected (Supporting Information, Figure S1).

### 3.2. Rheology of Monovalent Suspensions

Rheological testing provides the ability to probe the structure of fluids in out-of-equilibrium (flow) conditions. This is of relevance both in terms of elucidating their microstructure and interactions therein, but also towards designing processing applications, where flow often plays a decisive role on material structuring and performance [23,24,25]. Here, we focus on the ability of the suspensions to form gelled networks, and to assemble into liquid crystalline structures.

**Figure 4:**
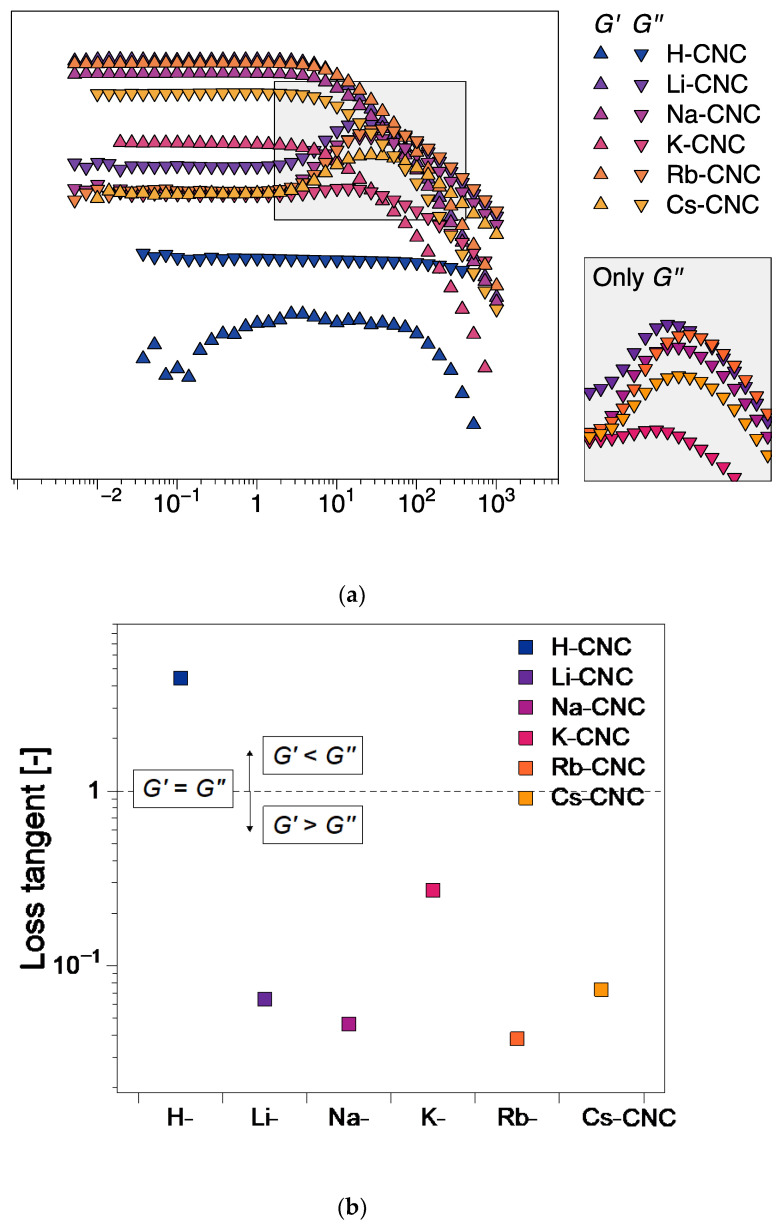
Oscillatory shear strain sweep tests for CNC suspensions presenting the (**a**) dynamic moduli profile against the shear strain amplitude and (**b**) loss tangent (tan δ = *G*″/*G*′) from the viscoelastic data in (**a**) as function of the type of counter cation. The oscillatory shear strain sweep tests were conducted at a fixed angular frequency of *ω* = 6.28 rad⁄s.

The linear viscoelastic properties from strain sweep tests (Figure 4a) showed a significant qualitative and quantitative difference between the protonated- and the alkali-metal cation-modified CNC suspensions. The protonated H-CNC showed a liquid-like behavior (loss modulus *G*″ higher than storage modulus *G*′), whereas the alkali metal cation modified CNC suspensions showed a gel-like behavior (*G*′ > *G*″). In addition, H-CNC dynamic moduli are at least one order of magnitude lower compared to those with the counter cations. Thus, H-CNC showed an inability to form a gelled network. The network-forming ability of the M-CNCs appeared unaffected by the increase in the cation size, and the quantitative and qualitative behavior of the dynamic moduli was mostly within the same magnitude range for all M-CNCs. However, an inspection of the loss tangent (tan δ = *G*″/*G*′ in the linear viscoelastic region) as a function of the counter cation type (Figure 4b) revealed that the K-CNC stood out from the M-CNCs. Whereas Li-CNC, Na-CNC, Rb-CNC, and Cs-CNC gelled networks (*G*′ > *G*″, tan δ < 1) are characterized by similar loss tangents, K-CNC has a higher loss tangent due to a lower storage modulus (Figure 4a). In addition to the linear viscoelastic behavior, all alkali counter cation CNC suspensions disclose a weak strain overshoot (WSO) behavior, whereby the storage modulus increases to a local maximum prior to the transition to the nonlinear viscoelastic regime (more details in Figure 4a). This type of material response is typically ascribed to the jamming and destruction of micro- to meso-ordered structures prior to yielding. However, K-CNC shows a significantly lower weak strain overshoot compared to the other alkali metal counter cations. The viscoelastic dynamic moduli and the WSO both suggest that the presence of K^+^ appears to have a disruptive effect on gel network formation among the alkali metal cation modified CNC suspensions.

**Figure 5:**
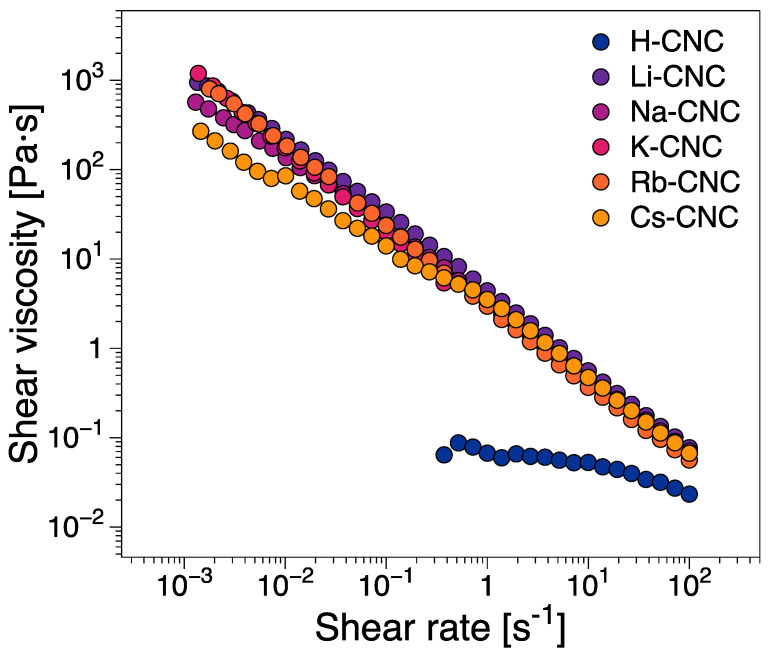
Steady shear viscosity functions of the CNC suspensions.

The rheo-PLI steady shear viscosity tests largely follow the behavior observed in the strain sweep tests. The steady shear viscosity functions (Figure 5) demonstrate the shear-thinning behavior of the CNC suspensions across the entire examined shear rate range. Quantitatively, the protonated CNC suspension viscosity is at least one order of magnitude below the alkali metal counter cation modified suspensions, with the latter showing similar viscosity magnitudes (Figure 5). Qualitatively, H-CNC appears to exhibit a zero-shear Newtonian plateau followed by a shear-thinning region. In contrast, Na-CNC, Rb-CNC, and Cs-CNC show, to a certain extent, evidence of a three-region viscosity behavior (Figure 5), a non-monotonic type of viscosity function comprising two shear-thinning regions separated by an intermediate plateau [19,20]. It has been shown that the three-regions correspond to shear-rate differentiated orientation, with the first shear-thinning region comprising an anisotropic ring in rheo-SANS [26] coupled with increasing anisotropy that increases with an increasing shear rate. A relatively constant anisotropy characterizes the intermediate region, and strong orientation in the flow direction in the last shear-thinning region [26]. Alhough three-region viscosity functions are often characteristic of liquid crystalline systems, they cannot be reliably employed to determine whether a suspension is biphasic or liquid crystalline [3]. The effect is most pronounced for Cs-CNC, where the three regions can be readily observed (Figure 5). This essentially means that, except for Cs-CNC, orientation dynamics are gradual throughout the shear rate range. The exact reasons why some biphasic and liquid crystalline suspensions show three region-viscosity regions and others do not, are yet to be elucidated. However, this is likely related to the structure and breakup of CNC aggregates during orientation in steady shear.

**Figure 6:**
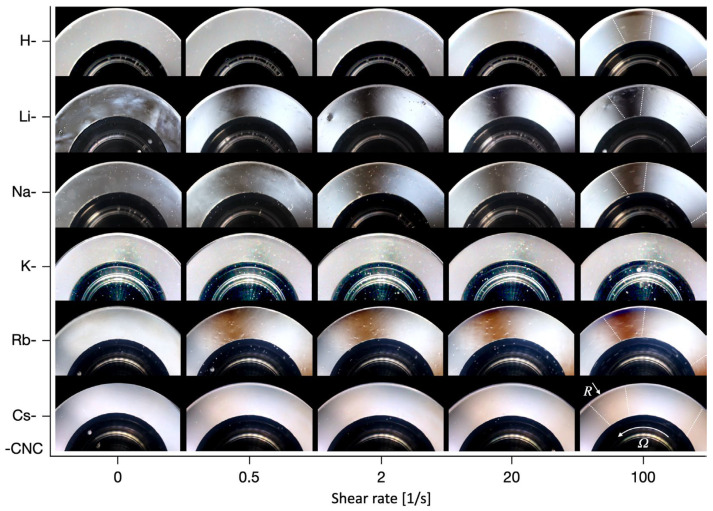
Birefringence patterns of CNC suspensions with respect to shear rate corresponding to the steady shear viscosity data in Figure 5. The white dotted lines at 100 s^−1^ are meant to approximate the location of the Maltese-cross fringes. The visualization background has been artificially replaced (black regions) to enhance contrast.

While viscosity functions provide a bulk measure of microstructural interactions, further information regarding the corresponding assembly phase and flow-induced orientation can be gained from examining the simultaneously recorded PLI (Figure 6). With an increasing shear rate, H-CNC exhibits a so-called Maltese-cross pattern at around 20 s^−1^. The Maltese-cross pattern is a consequence of the orientation of flow constituents in the flow direction (circular trajectory) as observed through a linear polarized light setup [27]. We note here that a 2 wt.% stock CNC suspension, having comparable PLI Maltese-cross patterns and within a range of shear viscosities and dynamic moduli that encompass the present H-CNC results, has been confirmed to be isotropic [18,28], within a range of preparation methods. Interestingly, considering that the observed Maltese-cross shows a brown coloring, we can conjecture that H-CNC retains the ability to assemble into flow-induced, likely-nematic structures, and, therefore, it is a biphasic system. In a slight contrast, Li-CNC and Na-CNC show evidence of biphasic structure in the form of partially light-blocking birefringence patterns even at rest (see 0 s^−1^ in Figure 6). An increase in shear rate results in an early orientation in the flow direction, as evidenced by the Maltese-cross pattern observable already at 0.5 s^−1^. K-CNC, previously shown to be disruptive of network formation, showed no evidence of self- or dynamically-assembled structures or orientation in the flow direction. We note that the K-CNC images have been edited to emphasize that although the presence of an image setup artefact may appear to be part of a Maltese-cross pattern, the artefact is identically present at all shear rates. In addition, there is no evidence of a four-quadrant symmetry characteristic of Maltese-cross patterns (Supplementary Information Figure S2). With an increasing counter cation size (Rb^+^ and Cs^+^), we note minimal birefringence patterns at rest (0 s^−1^) in contrast to Li-CNC and Na-CNC. However, colored Maltese-cross fringes were already visible at 0.5 s^−1^. This was most evident for Rb-CNC, where the brown-blue color palette (the blue can be observed at 100 s^−1^ in Figure 6 at the outer edge where the local shear rate is highest) is reminiscent of stock CNC biphasic suspensions in the 3–5 wt.% concentration range [18,19,20,28]. Cs-CNC showed a light-brown Maltese-cross (see also Figure S3), but with a more diffuse coloring, suggesting that the largest cation investigated may exhibit some self-assembly hindrance.

## 4. Discussion

**Figure 7:**
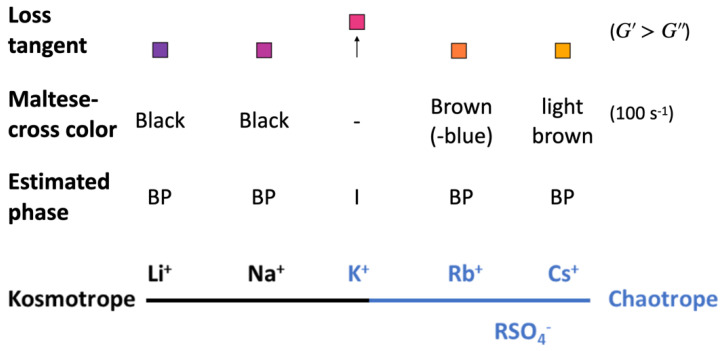
Ordering of the alkali metal cations, and sulfate ions in their kosmotrope and chaotrope character. Abbreviations: BP-biphasic, I-isotropic. The arrow indicates a higher loss tangent value for K^+^ than for other cations.

Counter cation exchange led to similar levels of the exchange rate (Figure 2) and to intact and identical physical appearance (Figure 3) for the alkali metal cations. This implies that any differences in the properties of these CNC suspensions should stem from the specific cation, its intrinsic properties, or the consequently altered CNC–CNC interaction. Li^+^ and Na^+^ are strongly hydrated kosmotropic species, whereas there is a switch in the sign of the Jones–Dole viscosity B coefficient, which is a measure of ion–water interactions when moving down in the alkali metals to K^+^, Rb^+^, and Cs^+^. That indicates a change into chaotropic, weakly hydrated ions [29,30]. Chaotropes can form direct ion pairs with other chaotropes, and kosmotropes with other kosmotropes. In contrast, chaotropes do not come into close contact with kosmotropes. The sulfate head groups in surfactants have been categorized as chaotropic [31], and, hence, should be able to form ion pairs with chaotropic ions such as Cs^+^, Rb^+^, and K^+^ in solution rather than with kosmotropic ions such as Li^+^ and Na^+^ (Figure 7). The ion pairs are less hydrated than separate ions and groups. According to Weißenborn and Braunschweig [32], the formation of the ion pair reduces the degree of dissociation of the pair components. This leads to a hypothesis that the alkali metal cation-sulfate ion pair is increasingly eager to form and be decreasingly hydrated in the order of Li^+^-RSO_4_^−^ < Na^+^-RSO_4_^−^ < K^+^-RSO_4_^−^ < Rb^+^-RSO_4_^−^ < Cs^+^-RSO_4_^−^ (Figure 7) [31]. In a previous report, the stability of sulfated Na-CNC in the presence of different monovalent first group cations was investigated [33]. The authors observed a critical aggregation concentration (CAC) for all ions, which originated from a reduction of effective Coulomb repulsions due to the presence of sulfate groups on the CNC surface. The CACs were in line with the Schulze–Hardy rule, as the critical aggregation concentration decreased with increasing counterion valence (Li^+^ > Na^+^ > K^+^ > Cs^+^). The behavior of the CACs was explained by matching affinities between the cation and the sulfate groups present at the surface of CNCs, confirming this above-mentioned hypothesis. However, we observed the K-CNC rheology and birefringence as a stand-out case within the series of alkali metal modifications, with dynamic moduli/loss tangent indicating a network disruptive effect compared to the other counter cations, whereas rheo-PLI showed the absence of self- or dynamically-assembled nematic/chiral nematic structures. For CNCs, the potassium grade has not been previously reported to stand out in the ability to self-assemble into the anisotropic phase in a static system [4]. However, for concentrated cellulose sulfate solutions that form liquid crystalline orders, the potassium counter cation has been reported to stand out in the inability to form spontaneously anisotropic phases, which was verified via observation of the birefringence patterns [34]. This ability was assigned to the structure-breaking character of K^+^, in contrast to the structure-making character of Li^+^ and Na^+^ ions [35]. Observations beyond K were not performed in that study. Progressing forward in the series to Rb^+^ and Cs^+^, we noticed that, at rest, liquid crystalline orders could not be detected, which fits well with the prediction that progressing further in the alkali metal series leads to increasing chaotropic character [36,37]. However, we observed that in flow, the nematic order is formed with Rb^+^ and Cs^+^, indicated by the appearance of a Maltese-cross. It is intriguing what makes the K^+^ system disrupt the flow assembly, but then continue with the Rb^+^ and Cs^+^. It has been reported that in a system of gelatin peptides, the alkali metal cations (Li^+^, Na^+^, K^+^, Rb^+^, Cs^+^) were used as counterions for a terminal carboxyl group [38]. The system viscosity decreased from Li^+^ to Na^+^, reaching a minimum with K^+^. Then, the viscosity again increased with Rb^+^ and Cs^+^. This complies to our observations of the disrupting power of K^+^, although we are unable to reveal the mechanism.

## 5. Conclusions

A facile method using excess of alkali metal cations led to the exchange of the counterion on CNCs. The modifications did not result in the loss of sulfate-half ester groups and represents a non-invasive way to modify CNC surfaces. Although full conversion of Na-CNC to H-CNC cannot be realized by HCl treatment, the exchange to Li^+^, K^+^, Rb^+^, and Cs^+^ can be achieved from the acid form, although the conversion to the H-form was only partial. This gives an indication that the exchange to the alkali metal cations does not require the intermediate acid form but can be achieved via the simple approach of excess cations in water suspension followed by dialysis. The cations with higher atomic numbers, i.e., larger ion sizes, generally tend to form stronger viscoelastic gels, as reflected in the behavior of the dynamic moduli of the metal cation CNCs. The choice of cation is potentially able to modulate the liquid crystalline behavior of CNC suspension in static and dynamic (flow) states, although the mechanism remains unelucidated.

## Figures and Tables

**Table 1:** Elemental composition (mg/kg) of the stock CNCs, and the H-CNCs treated with alkali metal salts.

	S	Li^+^	Na^+^	K^+^	Rb^+^	Cs^+^
CNC stock	7400 ± 300	8.1 ± 0.3	**4900 ± 200**	61 ± 2	7.4 ± 0.3	4.9 ± 0.2
H-CNC	7500 ± 300	15 ± 1	**2100 ± 100**	330 ± 10	146 ± 6	169 ± 7
Li-CNC	6800 ± 300	**1140 ± 50**	500 ± 20	290 ± 10	19.1 ± 0.8	12.5 ± 0.5
Na-CNC	7200 ± 300	13 ± 1	**4300 ± 200**	129 ± 5	26.7 ± 1.1	21 ± 1
K-CNC	6900 ± 300	12 ± 1	270 ± 10	**6600 ± 300**	5.9 ± 0.2	3.8 ± 0.2
Rb-CNC	6600 ± 200	20 ± 1	340 ± 10	89 ± 4	**15,600 ± 600**	9.0 ± 0.4
Cs-CNC	7000 ± 300	9.3 ± 0.4	290 ± 10	84 ± 3	9.6 ± 0.4	**25,000 ± 1000**

## Data Availability

Not applicable.

## Supplementary Materials

The following supporting information can be downloaded at: https://www.mdpi.com/article/10.3390/nano12183131/s1, Figure S1: Hydrodynamic dimensions of M-CNC water suspensions determined by dynamic light scattering; Figure S2: PLI visualization of K-CNC at 100 s^−1^ in a zoomed-out version proving the absence of a flow-induced Maltese-cross pattern; Figure S3: PLI visualization of Cs-CNC at 100 s^−1^ in a zoomed-out version proving the presence of a flow-induced Maltese-cross pattern; Table S1: Atomic composition (at.%) of the CNCs determined using XPS; Table S2: Comparison of XPS binding energies of metal sulfates from Wahlqvist et al. with the alkali metal cation modified M-CNC. Reference [22] was cited in the Supplementary Materials.
