# Supplementary material for: Dynamic and Static Assembly of Sulfated Cellulose Nanocrystals with Alkali Metal Counter Cations
Source: Nanomaterials (Basel). 2022 Sep 9;12(18):3131. doi: 10.3390/nano12183131 (PMC9502719; doi:10.3390/nano12183131)
Supplement: Supplementary file 1 [file nanomaterials-12-03131-s001.zip › nanomaterials-1888408-supplementary.pdf]

nanomaterials

MDPI

Supplementary Materials

Dynamic and Static Assembly of Sulfated Cellulose Nanocrystals with Alkali Metal Counter Cations

Patrick Petschacher [1], Reza Ghanbari [2], Carina Sampl [1], Helmar Wiltsche [3], Roland Kádár [2,4], Stefan Spirk [1,*] and Tiina Nypelö [4,5,*]

[1] Institute of Bioproducts and Paper Technology, Graz University of Technology, Inffeldgasse 23, 8010 Graz, Austria
[2] Department of Industrial Materials Science, Chalmers University of Technology, 41296 Gothenburg, Sweden
[3] Institute of Analytical Chemistry and Food Chemistry, Graz University of Technology, 8010 Graz, Austria
[4] Department of Chemistry and Chemical Engineering, Chalmers University of Technology, 41296 Gothenburg, Sweden
[5] Wallenberg Wood Science Center, Chalmers University of Technology, 41296 Gothenburg, Sweden
* Correspondence: stefan.spirk@tugraz.at (S.S.); tiina.nypelo@chalmers.se (T.N.)

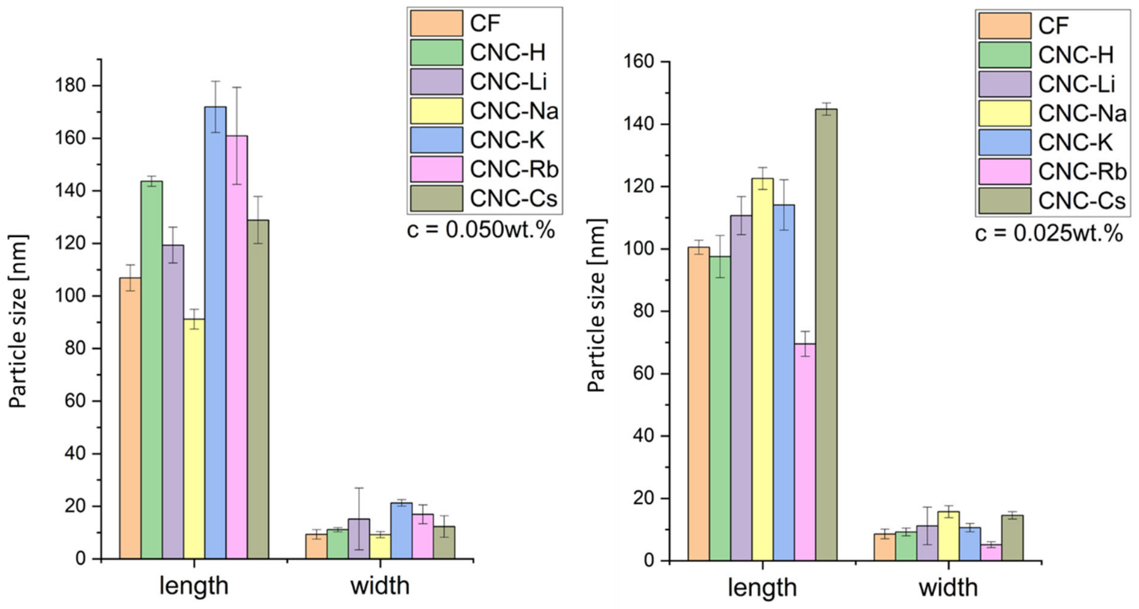

Figure S1. Hydrodynamic diameter of M-CNC water suspensions determined by dynamic light scattering.

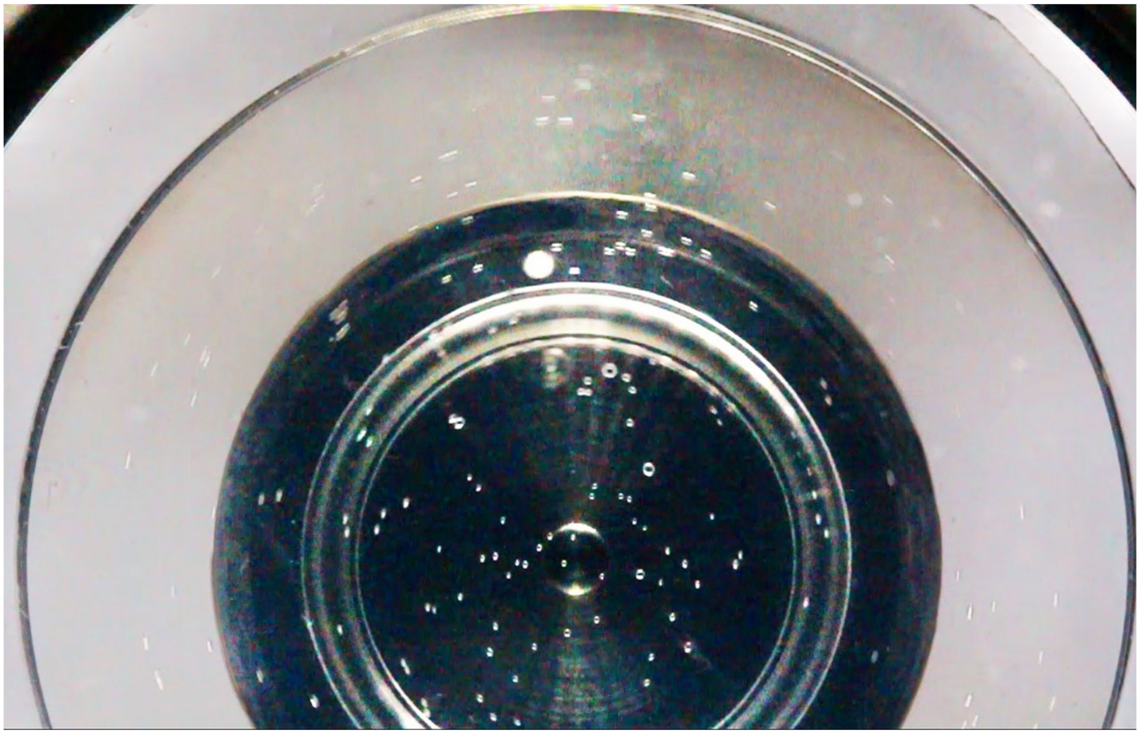

Figure S2. PLI visualization of K-CNC at 100 s^{-1} in a zoomed-out version proving the absence of a flow-induced Maltese-cross pattern.

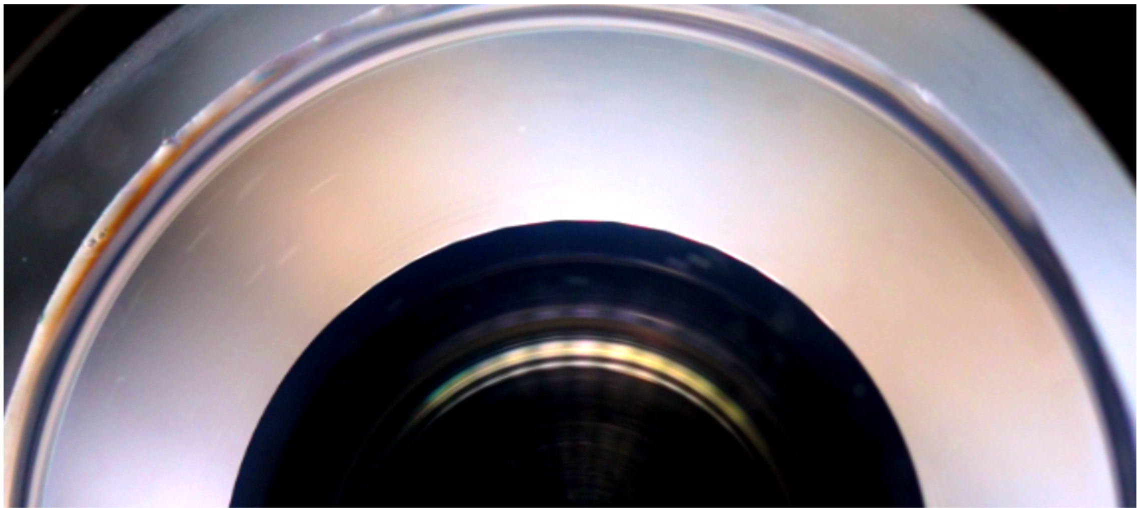

Figure S3. PLI visualization of Cs-CNC at 100 s^{-1} in a zoomed-out version proving the presence of a flow-induced Maltese-cross pattern.

Table S1. Atomic composition (at.%) of the CNCs determined using XPS.

	C1s	O1s	Si2p	S2p	Na1s	K2p	Rb3d	Cs3d
H-CNC	53.61	41.54	4.42	0.43				
Na-CNC	57.19	41.50		0.37	0.94	-		
K-CNC	58.65	40.77		0.35		0.23		
Rb-CNC	58.48	40.92		0.37			0.23	
Cs-CNC	57.87	40.49	1.01	0.42				0.20

Table S2. Comparison of XPS binding energies of metal sulfates from Wahlqvist et al. [1] with the alkali metal cation modified M-CNC.

	Li1s	Na1s	$K2p_{3/2}$	$Rb3d_{5/2}$	$Cs3d_{5/2}$
M_2SO_4 [eV]	56.1	1071.6	293.0	109.8	724.3
M-CNC [eV]	n.d.	1072.0	293.1	110.0	724.7

References

[1] Wahlqvist, M.; Shchukarev, A. XPS spectra and electronic structure of Group IA sulfates. *J. Electron Spectrosc. Relat. Phenom.* **2007**, *156*, 310–314. https://doi.org/10.1016/j.elspec.2006.11.032.
